# The Present State of Metropolitan Lying-In Hospitals

**Published:** 1888-03-31

**Authors:** Thomas Ryan

**Affiliations:** Secretary to St. Mary's Hospital, Paddington


### THE PRESENT STATE OF METROPOLITAN LYING-IN HOSPITALS.

By THOMAS RYAN, Secretary to St. Mary's Hospital.

THE sixth meeting of the present session of The Hospitals Association was held on the 14th inst., at St. Mary's Hospital, under the presidency of Dr. W. C. Grigg, Physician to Queen Charlotte's Hospital. There was a good attendance, consisting chiefly of ladies interested in the subject.

MR. THOMAS RYAN, Secretary to St. Mary's Hospital, read a paper, an abstract of which is given below, on

THE ORIGIN, HISTORY, WORK, AND PRESENT STATE OF METROPOLITAN LYING-IN HOSPITALS.

It is an opinion very widely held, that special hospitals are for the most part unnecessary, inasmuch as the work they undertake was, before they sprang into existence, performed by the general hospitals, and is indeed at present performed by them, at least as successfully, and, for reasons too obvious to call for mention, at a very much smaller cost. In addition to being unnecessary, they are also regarded as being positively harmful, in that they reduce the extent of the special departments at the general hospitals, and deprive them of an immense number of patients whose diseases would otherwise form the subject of clinical instruction to the students of the medical schools attached thereto. That the above opinions are, as a rule, borne out by the facts I shall not attempt to deny, but there are exceptions, it is said, to every rule, and I think it likely that there are a few exceptions to this. However that may be, it is no part of my business on this occasion to discuss the question; but I claim that, to the class of special hospitals of which my paper treats—namely, the lying-in hospitals—the objections do not apply.

It is an interesting fact that until the middle of the eighteenth century there were no medical charities in London of the class known as special hospitals. London was not the first town in the United Kingdom to possess a Lying-in Hospital, but was preceded in this particular by Dublin, where Dr. Bartholomew Mosse succeeded in opening a Lying-in Hospital in March, 1745. In 1749, four years after the founding of the Dublin Lying-in Hospital, London followed the example thus set, and a Lying-in Hospital was then instituted in Brownlow Street, Long Acre, under the presidency of the Duke of Portland, and was first opened for the reception of patients on December 7th of that year. There was no provision made for the attendance of patients at their own homes; but shortly after its foundation female pupils were received to be trained as midwives. In 1756 its name was altered, and it was thenceforth known as the British Lying-in Hospital. In 1849 it was rebuilt in Endell Street, St. Giles's, where it is at present situated. The hospital contains 28 beds.

The City of London Lying-in Hospital was established at London House, Aldersgate Street, on March 30th, 1750; Solnigsty Bethell, M.P., alderman of the City of London, being its first president. It was first commenced at London House, in hired apartments, and had then for its objects both the reception of in-patients, and the attendance of out-patients at their own homes.

Queen Charlotte's Hospital was instituted in 1752, and was thus the third in order of date among the Lying-in Hospitals of London. At the time of its foundation it was called the General Lying-in Hospital, and continued to be so called until Her Majesty Queen Charlotte became its patron in 1809, when its name was changed to that by which it is now known. It was the first Lying-in Hospital which combined the advantages of affording relief both to in and out patients, and the first also to take compassion on unmarried women with their first child; in addition to which the training of medical pupils and midwives was a recognised part of its work from the beginning. The Westminster Lying-in Hospital was instituted in 1768.

It is described in *Highmore's Public Charities* as being near Westminster Bridge, and is marked in maps of London of that time as standing about 300 yards from the bridge by the side of the main road (corresponding with the present Westminster Bridge Road) leading to St. George's Fields. The Hospital is now called the General Lying-in Hospital, having been built in York Road, Lambeth, at a short distance from its original situation, in 1830, at which time it was incorporated by royal charter. It contains twenty-four beds. These four are the only Lying-in Hospitals now existing which were established in the eighteenth century, but many others were founded at that time which have ceased to exist.

The work of the Lying-in Hospitals is three-fold: 1. The reception and delivery of lying-in women in the hospital. 2. The attendance of out-patients at their own homes by the hospital midwives. 3. The training of pupils in midwifery and midwifery nursing. The extent of the first two branches of their work will be seen from the following table, which gives the average annual number of in-patients and out-patients for the past three years at each hospital:

| | British. | City of London. | General. | Queen Charlotte's. | Total. |
|---|---|---|---|---|---|
| In-Patients ......... | 154 | 299 | 394 | 901 | 1,748 |
| Out-Patients ...... | 674 | 1,190 | 872 | 1,100 | 3,836 |

The work of the lying-in hospitals as midwifery training schools is shown in the following table, the figures representing the average annual number of pupils for the past three years:—

| | British. | City of London. | General. | Queen Charlotte's. | Total. |
|---|---|---|---|---|---|
| Midwives ............ | 10 | 18 | 16 | 23 | 67 |
| Monthly Nurses... | 55 | 94 | 57 | 144 | 350 |
| Total Female Pupils ........... | 65 | 112 | 73 | 167 | 417 |
| Medical Pupils .. | nil. | nil. | nil. | 60 | 60 |
| Total Pupils ... | 65 | 112 | 73 | 227 | 477 |

I propose now to make a few remarks on a subject which, in connexion with Lying-in Hospitals, is of greater moment and of more absorbing interest than any other. I mean the rate of maternal mortality among their in-patients. About ten years ago the death-rate of in-patients in the Lying-in Hospitals was, and had for some years been, very high. I will first give particulars of the mortality in these hospitals in the years referred to, accompanying them with some of the criticisms which were at that time publicly passed upon them. I will then place before you figures showing the mortality during the past eight years, when I have no doubt I may, with perfect confidence, leave you to form your own conclusions.

1. *Mortality of Mothers in Metropolitan Lying-in Hospitals*, 1870-79.—During those years the number of in-patients in four institutions was 11,807, and the number of maternal deaths from all causes 226, which gives a death-rate of 19 per 1,000. The highest death-rate was in Queen Charlotte's, where it was 24·4 per 1,000, and the lowest was at the General, whose rate of mortality was scarcely 13. Taking individual institutions and separate years, the highest rate reached was at the General, in 1877, where 9 patients out of 63 died, giving a rate of 143 per 1,000; and the best result was at the same hospital, in 1872, when among 311 patients there was not a single death.

2. *Remarks on the foregoing figures.*—Such a mortality as 19 per 1,000 admits of no satisfactory explanation, and I will not attempt to defend it, but will insert here the strictures which were passed upon it at various times in the decade during which it existed. I do this for two reasons: First, that I may be enabled presently to ask with more point, Where are the encomiums on the opposite state of things in the succeeding eight years? and secondly, for the purpose of calling attention to the false comparisons which these critics instituted between conditions essentially different. Miss Florence Nightingale, in a work on Lying-in Institutions, published in 1871, says: "Does not the whole of the evidence with regard to special lying-in hospitals lead but to one conclusion, viz., that they should be closed?" Elsewhere she compares the death-rate in lying-in hospitals with the mortality of women delivered at home, to the discredit of the former. Dr. Steele, in an essay dated June, 1887, which I have found in the "Journal of the Statistical Society," makes the following remarks: "Lying-in hospitals can hardly be said to have successfully fulfilled the objects of their promoters. The hospital which above all others has tended to throw discredit on lying-in institutions, by reason of its high mortality, is that known as Queen Charlotte's." He also makes comparison between the death-rate in lying-in hospitals, aud that of lying-in women at their own homes, with the same result as Miss Nightingale. The *Lancet* of 10th May, 1879, after giving some particulars of the mortality in Queen Charlotte's Hospital—which nothing but an overwhelming sense of its duty to the public would have induced it to publish—goes on to say: "These facts require explanation, and afford further grave evidence of the serious responsibility attaching to the maintenance of this institution as a hospital for the reception of in-patients." The *Lancet* also proceeds to make comparison between the results in the hospital as compared with that of mothers at their own homes.

3. *The Mortality of Mothers in Metropolitan Lying-in Hospitals*, 1880-87.—During these eight years the number of in-patients in the four hospitals has been 12,315, and the number of maternal deaths from all causes 138, giving a rate of mortality of 11·2 per 1,000. The highest death-rate has been at the City of London, where it has stood at 22·1, or nearly 6 per 1,000 worse than in the period already referred to, while the lowest rate of mortality was 6·1 per 1,000 at the General; Queen Charlotte's and the British being very close, with 8·5 and 9 respectively. If we leave out of the calculation the City of London, whose record is exceptional, we find that in the other three institutions there have been 9,924 deliveries, with 85 maternal deaths, which shows a death-rate of 8·56 per 1,000. The following table will show at a glance the old state of things side by side with the new:—

| | Deliveries. | Deaths. | Death-rate per 1,000. |
|---|---|---|---|
| 1870-1879 ...... | 11,807 ...... | 226 ...... | 19 |
| 1880-1887 ...... | 12,315 ...... | 138 ...... | 11·2 |

To have reduced the rate of mortality in their wards by nearly one-half, and this in spite of the fact that one among their number has unfortunately deteriorated, is, I should think, an achievement of which the hospitals have good cause to be proud, and which a fair critic ought to have brought prominently to the notice of the public, that the institutions might have reaped, in the form of increased public support, the reward which they deserve, just as in the dark days to which I have above alluded their delinquencies were made much of, with the result that there was a great falling off in the number of contributors to their funds. Of course I do not for a moment mean to say that Miss Nightingale and Dr. Steele should have written a book and an essay respectively vindicating the claims of the lying-in hospitals to the confidence and support of the public; but I do say that a medical paper such as the *Lancet*, having felt it its duty to publish the shortcomings of the lying-in hospitals, should have been just as ready, nay—even more so—to noise abroad the result of the past eight years. Perhaps the *Lancet* thinks, with some other critics, that its office is to expose defects and not to point out merits.

In a preceding part of this paper I mentioned that the causes of the high mortality which formerly obtained are now well known. I will briefly mention what those causes were. There is no doubt, in my mind, that they all arose from the fact that the entire care of the patients under the visiting physicians was committed to an unscientific person—to wit, a matron—who was not always even a hospital-trained nurse. Queen Charlotte's Hospital was the first among the lying-in hospitals to abolish this system, and to appoint a fully-qualified medical man as resident medical officer. From that day dates the improvement which culminated in the successes I have just mentioned. The adoption of the principles of Sir Joseph Lister, to which the Rotunda Hospital in Dublin, and Queen Charlotte's and the General in London—I am speaking from my own knowledge, which does not extend to the other two London hospitals—attribute their diminished mortality, could never have been successfully accomplished unless this change in the administration had been made. I have no right to say that the improvement in the rate of mortality at the General has also resulted from the appointment of a resident medical officer; but I can say that the worst results in its history were in the years 1877, 1878, and 1879, that a resident medical officer was appointed in 1880, and that the mortality since has been less than ever it was before. It is impossible to conceive a testimony more creditable to the medical officers and to the nursing staff of these charities than this—that their efforts have succeeded in establishing these institutions in a position more worthy of public recognition and support than they ever occupied before. Such efforts are worthy of the cause in which they are made, and surely a better cause does not exist than the succour of indigent women at a time when they stand most in need of our warmest sympathies and of our kindliest offices.

Miss Wilson (honorary secretary to the Midwives' Institute) commenced the discussion by declaring that the chief difficulty experienced by women who desired to be trained as midwives arose from the high fees charged by the various lying-in hospitals for pupil nurses. The demand for midwives was never greater than at present, nor the standard higher; but the fees were altogether prohibitory. It was true that the fees at certain workhouse infirmaries, like those at Brownhills, Liverpool, where £10 was charged for three months' training, and at Crumpsall, Manchester, where the fee was the same, were, therefore, reasonable, having regard to the fact that they were inclusive of board and lodging. The experience of the Institute, as well as of the Workhouse Nursing Association, proved that the demand for midwives was always greater than the supply, and that a reduction in the fees charged to pupil nurses by the authorities of lying-in hospitals was imperatively called for.

Miss Paget referred to the Bill before Parliament for the registration of midwives, and said that the present abuses would never be redressed until registration was established upon a proper basis. In reply to a remark that one register for all nurses would be an evil, she subsequently pointed out that she was not referring to the British Nurses Association or its scheme, which, whether it procured a royal charter or not, would do no good to midwives in any way.

Mr. F. Gardiner, secretary of the British Lying-in Hospital, said that the fee of ten guineas was not prohibitory at his institution. His experience was that matrons who were trained midwives did very good work, and that a house-surgeon was in such cases by no means necessary. There was no house-surgeon at the present time at the British Lying-in, or the City of London Lying-in Hospitals, and both institutions showed excellent results.

Mr. Burdett pointed out that medical students had few opportunities to qualify themselves for the work which would be entailed upon the house-surgeon of a lying-in institution, and that for this reason, except in a few cases, where special training and experience had been obtained, a trained and certificated midwife might well be placed in charge of these small hospitals. It was essential that this class of hospital should contain but few beds, and the absence of a house-surgeon had the advantage of ensuring that the honorary medical officers must be regular in their attendance and devoted to their work. He pointed out that the mortality statistics quoted by Mr. Ryan referred chiefly to a period when the sanitary condition of this class of hospital, and especially that of Queen Charlotte's Lying-in Hospital, left much to be desired, and the statistics of the last few years would show totally different results to those brought out in the paper. Mr. Ryan, as a late officer of Queen Charlotte's Hospital, not unnaturally held a brief for that institution, and it was desirable, therefore, to point out a serious omission in the paper. It would have been more satisfactory had he shown the infant mortality, which was stated to be higher at Queen Charlotte's than elsewhere. Alluding to the remark of Miss Paget on registration, Mr. Burdett stated that it would be a bad day for nursing should it ever come to pass that there was only one register. Indeed, the registration of nurses was a two-edged weapon which might cut more ways than one. It was satisfactory to know that the managers of the nurses' training schools throughout the country were fully alive to this danger, and that there was a general feeling in favour of each school examining and registering its own nurses, which would give the maximum of protection to the nurses and the public. It would not be fair for the nurses of a leading school to be rendered liable to suffer discredit from the action of the least reputable, as they most certainly would do should registration ever take the form of an independent and separate organisation, distinct from the nurses' training schools. Let each hospital register its own nurses, and send them out to nurse the public, who would thus learn to appreciate the difference between sound training and the reverse.

Mr. Hunt, Dr. Phillips, and one or two others having spoken,

Dr. Grigg closed the discussion. He said that if the Midwives Bill were to become law, the fees paid by midwives would probably be reduced. He deprecated Miss Wilson's advocacy of the penal clauses, as he held that stringent legislation of this character was opposed to the feeling of Parliament, and that the Bill would be imperilled if this clause were made too drastic. He suggested that they should be left out of the Bill altogether, because he was certain that the necessary discipline could be enforced afterwards by private pressure. He compared the work of Queen Charlotte's to that of the City of London Lying-in Hospital as proof positive that a house surgeon was essential to successful treatment. All the house surgeons at Queen Charlotte's Hospital had been men of large capacity and great experience, and he did not know of an instance where a house-surgeon had been appointed unless he had had special experience in this branch of the profession.

